# Editorial

**Published:** 1866-06

**Authors:** 


### Editorial.

### AMERICAN MEDICAL ASSOCIATION.

### ANNUAL MEETING, AT BALTIMORE, MD., MAY 1ST, 1866

This Association met in Concordia Hall, on Eutaw Street, Baltimore, and was called to order by the President, D. Humphrey Storer, of Boston, at 11 A.M., of May 1st, 1866. The number in attendance was large, and embraced delegates and members from all parts of the country. An appropriate prayer was made by Rev. Dr. SPEER, of Baltimore; after which, Dr. C. C. Cox, Chairman of the Committee of Arrangements, in an eloquent address, tendered the members of the Association a cordial reception, both by the profession and the citizens of Baltimore. The Committee also reported the names of such delegates and members as had been registered up to that time.

The election of members by invitation being in order, Dr. Jas. E. Rives, of Fairmount, West Virginia, was elected, and the rule suspended to enable Dr. C. C. Cox, of Baltimore, to make a statement in relation to the expulsion of Dr. M. A Pallen, during the annual meeting in Boston the preceding year. Dr. Cox stated that, since the last meeting, Dr. Pallen had placed in his hands such evidence and documents as were entirely satisfactory to his own mind, that the statements on which Dr. Pallen was expelled were without foundation; and, as he had moved the expulsion of Dr. Pallen last year, he was desirous of embracing the first opportunity to restore him to his former position. He therefore moved that the papers and documents in his possession be referred to the Committee on Ethics, with instructions to report as early as practicable. After a little discussion the motion was adopted, and the committee made to consist of Drs. Hooker, of Connecticut, Brinsmade, of New York, and Davis, of Illinois. At a subsequent part of the same morning session, the committee made the following report, which was unanimously adopted:—

"The committee, to whom were referred the papers in relation to the expulsion of Dr. Montrose A. Pallen, at the meeting of the Association in Boston, respectfully report that they have examined the documents and evidence referred to them, embracing papers endorsed by Lieut.-Gen. U. S. Grant, the Vice-Consul of the United States at Montreal, and many citizens of Missouri, and are fully satisfied that the statements on which his expulsion was based were entirely unfounded, and, therefore, regretting the injustice done, both to Dr. Pallen and the Association, we recommend the adoption of the following resolution:—

"*Resolved,* That the preamble and resolution adopted by the Association at the Annual Meeting in Boston, June, 1865, expelling Dr. Montrose A. Pallen, be hereby rescinded, and that he be restored to his previous membership in the Association."

Dr. Pallen, who was present as a delegate from the St. Louis Medical Society, was then introduced and received the cordial greeting of the Association.

Dr. D. H. Storer, of Boston, the President of the Association, read his annual address, which was listened to with marked attention. It related chiefly to a subject that has elicited much discussion, both in this country and Europe, *viz.*, how far specialties and specialists should be encouraged and recognized in our profession. The thanks of the Association were tendered to Dr. Storer for his address, and a copy requested for publication in the Transactions.

The whole list of special committees was then called, and such reports as were ready, together with such volunteer communications as were presented, were referred to the appropriate Sections for consideration.

On motion, Dr. E. Brown-Séquard was invited to address the Association, on the Treatment of Nervous Disorders, at 11 o'clock A.M. on Wednesday.

Dr. Jewell announced that Dr. Marsden, of Quebec, Canada, was present, and, on motion, he was elected a member by invitation, and requested to take a seat on the platform.

The Association then adjourned until 9 o'clock A.M. of Wednesday, the afternoon being occupied by the Sections in hearing and discussing such subjects as had been referred to them.

WEDNESDAY, MAY 2D—SECOND DAY.

The Association was called to order at 9½ o'clock A.M., the President, D. H. Storer, of Boston, in the Chair.

After some business notices by Dr. Cox, Chairman of the Committee of Arrangements, Drs. J. A. Reed, W. Whitridge, L. M. Eastman, of Baltimore, and Dr. Peter Parker, of China, were elected as members by invitation.

The reports of the Committee on Publication and of the Treasurer were read, accepted, and ordered on file. These reports showed that the publication of the last volume of Transactions had left the treasury in debt to the publishers to the amount of $404.52. This was owing, in part, to the expensive character of many of the illustrations, especially those accompanying the prize essay.

The Association then took a recess of fifteen minutes, to enable the delegates from each State to select one of their own number, to constitute a committee whose duty it should be to nominate officers for the ensuing year.

After the recess, the regular order of business being the reports of standing committees, the Committee on Medical Education was called, when Dr. S. D. Gross, of Philadelphia, Ch'n. of the Committee, stated that no report had been prepared, partly, at least, from the fact that the members of the Committee resided so far apart that no conference could be had on the subject referred to them.

Dr. Charles A. Lee, of New York, Chairman of the Committee on Medical Literature, proceeded with the reading of his report, but before completing it the hour of 11 o'clock A.M. arrived, which had been fixed for hearing an address from Dr. E. Brown-Séquard. The further reading was suspended, and the report referred to the Committee on Publication. The report of Dr. Lee was lengthy, and contained some topics and observations of doubtful propriety. It should have received a more careful consideration before its reference for publication.

Dr. Brown-Séquard took the platform and entertained the

24

Association for one and a-half hours, in a most interesting and profitable discussion of the subject of Nervous Affections and their Treatment.

On motion, the thanks of the Association were tendered to Dr. Brown-Séquard, and a copy of his lecture requested for publication.

The hearing of reports from standing committees was resumed, and Dr. E. Elliott presented the report of the Committee on Prize Essays, by which it appeared that four essays had been presented to the committee. The committee recommended two of these for prizes, *viz.:*—one, "On Health in Country and City," by W. F. Tounes, M.D., of New York; and one "On Digitaline, its Properties chemically and therapeutically considered," by S. R. Percy, M.D., of New York. The report of the committee was accepted and referred to the Committee of Publication.

Dr. W. Hooker, of Conn., Chairman of the Committee on Ethics, presented a report on the subject of Specialties, which was referred to the committee at the previous annual meeting. After hearing the report, its consideration was made the special order for 9½ o'clock A.M., on Thursday, and immediately after its disposition Dr. Marsden, of Canada, was invited to address the Association on the subject of Cholera and Quarantine.

The presentation of resolutions being in order, Dr. W. Hooker offered a resolution, declaring that no paper should be received by the Association, unless completed so as to be ready for direct transmission to the Publication Committee, without retention or revision by the author; which was adopted.

Dr. J. F. Hibbard, of Ind., presented a preamble and resolution, approving of the action of a convention of delegates from Western Colleges, recently held in Cincinnati, proposing a longer annual lecture term and a more uniform rate of fees, the consideration of which was made the special order for 11 o'clock A.M. of Thursday.

Resolutions of respect and condolence were offered in regard to the late Dr. James Couper, of Delaware, Dr. Jos. M. Smith, of New York, and Dr. D. L. McGugin, of Iowa, recently deceased, which were unanimously adopted.

On motion of Dr. M. K. Taylor, of Iowa, a committee of one from each State represented was appointed to prepare a memorial to Congress, asking for an appropriation sufficient for the early publication of the medical and surgical history of the war for subduing the recent rebellion, as prepared under the direction of the Surgeon-General of the U.S.A.

The Association then adjourned, to 9 o'clock A.M., of Thursday; the afternoon being devoted to scientific investigations in Sections.

THURSDAY, MAY 3D—THIRD DAY.

The President, Dr. D. H. Storer, called the Association to order at 9 o'clock A.M.

After some miscellaneous business, the special order for 9½ o'clock was taken up. A majority and minority report on the subject of *Specialties* were read and discussed at considerable length; when, on motion, the whole subject was postponed until the next annual meeting.

Dr. Marsden, of Canada, then occupied the attention of the Association for one and a-half hours, in giving a detailed description of his plan of quarantine, designed to prevent the introduction of cholera into the country. The views of Dr. Marsden are founded on the supposition that cholera is contagious, and can be prevented wholly by rigid quarantine and isolation. The subject was made the special order for consideration at 5 o'clock P.M.

Dr. N. S. Davis, Chairman of the Committee on Nominations, asked and obtained leave to report, as follows:—For President of the Association, H. F. Askew, of Delaware; For Vice-Presidents, Wm. K. Boling, of Tenn., J. C. Hughes, of Iowa, H. I. Bowditch, of Mass., and T. C. Brinsmade, of New York; Permanent-Secretary, W. B. Atkinson, of Penn.; Assistant-Secretary, W. W. Dawson, of Ohio; Treasurer, Caspar Wistar, of Penn.; For Committee of Arrangements, J. A. Murphy, James Graham, R. R. McIlvaine, J. P. Walker, W. T. Brown, and W. B. Davis, all of Cincinnati, O.; Committee on Publication, the same as at present; Committee on Education, S. D. Gross, D. F. Condie, John Bell, of Philadelphia, H. J. Bigelow, of

Boston, and Chas. A. Pope, of St. Louis; Committee on Medical Literature, A. C. Post, James Anderson, A. D. Noyes, T. G. Thomas, and Stephen Smith, all of New York; Committee on Prize Essays, F. Donaldson, Jos. Simpson, C. C. Cox, E. Warren, and W. C. Van Bibber, all of Baltimore. The Committees on Climatology and Epidemics, and on American Medical Necrology, were recommended to be continued, with only a few changes and additions.

The next annual meeting was recommended to be held in Cincinnati, on the first Tuesday in May, 1867.

On motion, the report of the Nominating Committee was accepted and adopted by a unanimous vote.

The Association then went into Committee of the Whole, for considering the preamble and resolution concerning the action of Western Medical Colleges on the subject of extending the lecture terms and fees; Dr. J. F. Hibbard, of Indiana, in the Chair. A very interesting discussion ensued, which was participated in by Drs. Wright, of Cincinnati, Storer, of Boston, Hooker, of New Haven, Davis, of Chicago, and Allen and Anticell, of Washington. Dr. Davis offered the following, as a substitute for the preamble and resolution under discussion:—

*Resolved*, That this Association earnestly request the Medical Colleges of the country to hold a convention for the purpose of thoroughly revising the present system of medical college instruction, and that a committee be appointed to aid in carrying the resolution into effect.

The substitute was adopted, and the Committee of the Whole rose and reported.

On motion, the action of the Committee of the Whole was concurred in by the Association, and the President appointed Drs. N. S. Davis, of Chicago, W. Hooker, of New Haven, S. D. Gross, of Philadelphia, M. B. Wright, of Cincinnati, G. C. Shattuck, of Boston, the committee called for by the resolution of Dr. Davis.

After some miscellaneous business, the Association adjourned to 5 o'clock P.M.

*Afternoon Session.*

At 5 o'clock P.M., the members of the Association re-assembled, and were called to order by the President.

On motion, the Association went into Committee of the Whole, on the subject of Dr. Marsden's plan of quarantine, Dr. N. S. Davis being called to the Chair.

Dr. L. A. Sayre, of New York, commenced the discussion, strongly advocating the doctrine of contagion, and the system of quarantine recommended by Dr. Marsden. Drs. M. L. Linton, of St. Louis, and J. N. Bell, of Brooklyn, followed upon the opposite side. Drs. John L. Atlee, of Lancaster, Chas. A. Lee, of New York, and Marsden, of Quebec, supported the doctrine of contagion and quarantine; while Dr. Wilson Jewell, of Philadelphia, sustained the opposite side with ability. Dr. King, of Pennsylvania, offered a resolution, endorsing the plan of quarantine, and urging its adoption on Congress. But without coming to any conclusion, the committee rose and reported progress.

The Association then adjourned to 9 o'clock A.M. of Friday.

FRIDAY MORNING—FOURTH DAY.

The Association was called to order at 9 o'clock, Dr. D. H. Storer, President, in the Chair.

The proceedings of the preceding sessions of the Association were read by the Permanent-Secretary and approved.

Some miscellaneous business was transacted, amongst which was the adoption of resolutions of thanks to the President, to the Committee of Arrangements and Profession of Baltimore, and to the Governor of the State and Civic Authorities of the City, for the very hospitable manner in which the Association had been received and entertained during the present annual meeting.

The Secretaries of the several Sections reported in order the doings of each Section; and their reports were severally accepted and referred to the Committee on Publication.

The Association then went into Committee of the Whole, for the further consideration of the resolution of Dr. King, commending the plan of quarantine proposed by Dr. Marsden, and Dr. N. S. Davis was called to the Chair.

Dr. Birge offered a resolution, as a substitute for the one offered by Dr. King, declaring the necessity of efficient measures for preventing the spread of cholera, and asking Congress to take such measures as would test the value of the plan of quarantine proposed by Dr. Marsden. An interesting discussion followed, which was participated in by Drs. Bell, of Brooklyn; Sayre, of New York; Hooker, of New Haven; D. H. Storer of Boston; A. C. Post, of New York; N. Pinckney, of U.S. N., Marsden, of Quebec; and Anderson, of New York.

On motion, the resolution and substitute were laid upon the table. The Committee of the Whole then rose and reported the action taken, which was concurred in by a vote of the Association.

After some items of miscellaneous business, the Association adjourned to meet in Cincinnati, on the first Tuesday in May, 1867.

Thus ended the doings of the Nineteenth Annual Meeting of the Association, so far as the general sessions were concerned, but the principal work of scientific interest and importance was transacted in the several Sections. We were not able to preserve a list of all the papers and reports that were read in the Sections and referred to the Committee of Publication, but they were sufficient to make a good-sized volume, and some of them were papers of decided merit. Among them, was a report on diphtheria, by Dr. Holton, of Vermont; one on spotted fever, or cerebro-spinal meningitis, by Dr. Levick, of Pennsylvania; one on the etiological and pathological relations of epidemic erysipelas, spotted fever, and diphtheria, by Dr. Davis, of Illinois; and one on alcohol and its relations to man, by Dr. J. R. W. Dunbar, of Maryland.

The social interests of the Association were well provided for. The first evening was occupied by an exceedingly pleasant promenade concert in Concordia Hall. The Hall itself was elegant, the music excellent, and the free social greeting and intercourse all that could be desired.

The second evening was occupied by cordial and elegant receptions at the residences of Drs. C. C. Cox, J. Simpson, and T. E. Bond. On the third evening, the Mayor and Council of the City of Baltimore gave the Association a very bounteous and hospitable entertainment, which was accompanied, as usual, by toasts and speeches, until a late hour. The occasion was highly enjoyed by all, and was quite free from those excesses which sometimes mar such public festivities. On the afternoon of the fourth and last day, the members of the Association, with their ladies, were provided with an excursion to Annapolis and a reception by the Governor of Maryland. This was, in all respects, one of the most pleasant seasons that the members of the Association have ever enjoyed. The day was fine; the boat in excellent order, with a splendid band of music from Fort McHenry; the company enlivened by the presence of many ladies of the highest social qualities; and the reception by Gov. Swan, with the interchange of sentiments between him and Dr. Cox, as the representative of the Association, all contributed to make the occasion one long to be remembered with great pleasure. Altogether, the influence exerted by this annual meeting of the Association, at this crisis in the history of our country, cannot fail to be of a permanently beneficial character.

A more extended notice of the work done in the several Sections, must be reserved until our next issue.

---

NEW BOOKS.—We have space only to acknowledge the reception of the following new works:—

RECENT ADVANCES IN OPHTHALMIC SCIENCE. By H. W. WILLIAMS, of Boston. TICKNOR & FIELDS Publishers. 1866.

For sale by J. R. Walsh.

ASIATIC CHOLERA. By F. A. BURRALL, M.D. Published by WM. WOOD & Co., 61 Walker St., New York. 1866.

For sale by Keen & Co., 148 Lake St., Chicago.

TRANSACTIONS OF THE AMERICAN MEDICAL ASSOCIATION, Volume 16, for 1865.

A large-sized, neatly-published volume, the contents of which shall be noticed more fully hereafter.

### THE AURORA HOMEOPATHIC QUACKS TREATED TO AN "ALLOPATHIC DOSE OF FACTS."

BY D. W. YOUNG, M.D., of Aurora, Ill.

---

The following spicy correspondence is taken from the Aurora *Beacon:*—

At a recent meeting of the Aurora City Medical Association, DR. YOUNG said he had now under treatment one of the worst cases of mercurial sore mouth that he had seen in ten years. The patient had been treated for eight weeks by one of the Aurora homeopathic doctors for a simple ailment, and had come out of the conflict minus $80.00 in cash, and with a magnificent *calomel sore mouth.* "He presumed, of course, the remedy had been given under the sanction of homeopathic law." In this case they are estopped from resorting to the usual homeopathic dodge of charging it to the account of previous medication, for the reason that the patient is a young person that had never been subject to medical treatment before, and therefore it is unquestionably a case of original homeopathic "mercurius." He thought it undeniable evidence of "Medical Progress." He said if he should find as bad a case of mercurial salivation, resulting from medical treatment, in the hands of a regular physician for such a disease, he should certainly recommend a prosecution for mal-practice.

To which the Homeopaths replied as follows:—

NOTICE.

*Editor Beacon:*—We, the undersigned, believing that in the report of the A. C. M. Association, published in your issue of last week, Dr. Young attempted to vilify and slander Homeopathy, and believing his assertions to be *false in every particular*, challenge him to publish the name of the physician and patient, for the benefit of the public and in vindication of his own honor. (Signed,) A. R. BARTLETT, M.D.,
F. H. VANLIEW, M.D.,
A. W. WOODWARD, M.D.,
W. M. L. FISK, M.D.

To which request Dr. YOUNG replied promptly as follows:—
To A. R. Bartlett, M.D., F. H. VanLiew, M.D., A. W. Woodward, M.D., and W. M. L. Fisk, M.D., Homoeopathic Quacks of Aurora.

*Gentlemen:*—Your communication published in the Aurora *Beacon*, April 12th, commenting upon my report to the Aurora City Medical Association of the case of "Magnificent Homeopathic calomel sore mouth," is before me. In reply allow me to say that it affords me unbounded pleasure to comply with your petition or challenge so far as to publish for "the public benefit," the name of the homeopathic physician who treated the patient (his name is Fred. H. VanLiew) and regret exceedingly that professional honor precludes me from publishing the name of the patient. But in lieu thereof I will publish the following certificates substantiating my statement:—

This may certify that I went with Mr. —, a friend and messmate of mine, who was suffering severely with a sore mouth, to Dr. D. W. Young's office, and while there heard him tell Dr. Young that he had been treated by Dr. Fred. H. VanLiew for eight or nine weeks; that while he was taking VanLiew's medicine his mouth and gums became very sore, teeth loose, breath fetid, and had a peculiar metallic taste in his mouth. When he spoke to VanLiew concerning his sore mouth, he told him *it was scrofula coming out.* I know he druled constantly, and was compelled to spit nearly all the time.

G. M. Caskey, Painter at Car Shops.

April 13, 1866.

I hereby certify that I was present in Doctor Young's office and heard the above conversation. I saw the man's mouth, and know that he druled badly and spit almost constantly. I have no doubt but it was a pure case of calomel sore mouth.

John Green, Medical Student with Dr. Young.

I do confess, gentlemen, that I am surprised at your pretended ignorance in attempting to deny that such cases do occur in your practice, *when you know them to be of quite frequent occurrence.* Every physician who has practiced medicine in Aurora any length of time, and met your patients, knows this to be the case. I did not report this case because of their rare occurrence, but because of its severity. At the last meeting of the Fox River Valley Medical Association, Dr. Hard reported a similar case that occurred under the treatment of one of your number. Please challenge him, and I have no doubt he will publish the name of the physician for the "public benefit." As further proof that you do use calomel, I publish the following testimony of Dr. Taylor, the celebrated English chemist, before the select committee of Parliament. He said: "I detected, in six powders, that three contained sugar of milk, and three contained morphine and *calomel.* The powders were numbered to be taken on certain nights, and in every other powder there was sugar of milk, and in every other powder morphine and *calomel.*"

I am, gentlemen, with great respect, your obedient servant,

D. W. Young, M.D.

---

Philadelphia, *Jan. 20, 1866.*

Dear Sir:—Your attention is invited to the following letter and prospectus. Should the enterprise named therein commend itself to your support, we shall be happy to forward your name as a subscriber. Very respectfully,

Your obedient servants,

J. B. LIPPINCOTT & Co.

---

Royal Library, Windsor Castle,

*21st December, 1865.*

My Dear Sir:—I send you a prospectus of a work of great interest and importance, which I believe may attract as much attention in America as it can on this side of the Atlantic: for the name of Leonardo da Vinci belongs to the world; and this record of his profound studies in science, and his consummate skill in art, concerns all who care to know how human civilization has reached its present elevated point.

The MS. which I am about to publish has belonged to the Royal Collections ever since the time of Charles II. But, by a most remarkable accident, it was laid aside and totally forgotten for full 90 years after it had been purchased. Since 1763, when it was discovered in an old bureau at Kensington Palace, it has been seen by a few persons only; because it formed a part of the private collections of the Crown. But all who were able to appreciate it joined in the opinion I have quoted in my prospectus from the writings of the celebrated Dr. Wm. Hunter.

This great surgeon proposed to publish some portions of the MS. in *fac-simile*, but was prevented by death. At a later time, Mr. Chamberlaine, one of my predecessors in the care of the Royal Collection of Prints and Drawings, entertained the same design, and did engrave six plates, when he was stopped by the prodigious expense of the undertaking. The discovery of the process of photolithography renders it now comparatively easy to reproduce an exact copy of each line and touch of the great artist. And when it is considered that this MS. records the first discovery of the circulation of the blood, and fills up a great blank in the history of anatomical science, while the drawings are perfect models for the student of art, I cannot but think my enterprise will be warmly supported.

Her Majesty the Queen has headed the subscription list with an order for ten copies, and their Royal Highnesses the Prince of Wales (to whom the work will be dedicated), the Prince of Prussia, Prince Alfred, and Prince Louis of Hesse, have also given me their names; I have also, almost before I have announced my plan, received the subscriptions of the British Museum Print Room, the Department of Science and Art, and of several both of the nobility and gentry, and scientific and artistic men, who desire my series.

I need not say that I shall welcome most warmly any support you can procure me in the United States; and so

I remain, my dear Sir,

Yours most truly,

J. B. LIPPINCOTT, ESQ. B. B. WOODWARD.

---

PRIVATE PROSPECTUS.

The Anatomical Drawings and Writings of Leonardo da Vinci are among the choicest treasures in the Royal Library at Windsor Castle. They are contained in about two hundred detached leaves of note-books, and appear to be the records of studies commenced as his needful training in art, and would, perhaps, have been employed in the great Treatise on Painting which he afterward projected. But he carried his researches far beyond what was required for these purposes, and arrived at results which entitle him to a place in the foremost rank of discoverers in this branch of science. Vasari, after describing (with a little pardonable inaccuracy) these works, reports the opinion entertained of them in his day. "It appears," he says, "almost incredible that this sublime genius could at the same time discourse, as he has done, of art, and of the muscles, nerves, veins, and any part of the frame, all treated with equal diligence and success." And Dr. Wm. Hunter, who saw them in the last century, in the Royal Collection, thus expressed his appreciation of them:—"When I consider what pains he has taken upon every part of the body, the superiority of his universal genius, his particular excellence in mechanics and hydraulics, and the attention with which such a man would examine and see objects which he was to draw, I am fully persuaded that Leonardo was the best anatomist, at that time, in the world."

These drawings and writings it is now proposed to publish in *fac-simile*, on account of their combined artistic and scientific value. And Her Majesty the Queen has been graciously pleased to permit their publication in the interests of these studies.

The whole work will consist of about two hundred and fifty plates, in folio, with the text of the MS. printed in full, an English and a French translation, and all needful notes and elucidations.

Mr. Panizzi, Principal Librarian of the British Museum, has undertaken to superintend the text, and Dr. Sharpey, Sec. R. S., Prof. of Anatomy and Physiology in University College, London, will assist in the preparation of the scientific commentary.

It will be issued in twenty parts, at the price of one guinea each, and the publication will commence early in the year 1866.

B. B. WOODWARD.

ROYAL LIBRARY, WINDSOR CASTLE,

*2d Oct., 1865*

—

Subscriptions received by J. B. LIPPINCOTT & CO.,
*715 & 717 Market St., Philadelphia.*

---

THE RINDERPEST.—Most of our daily papers are filled with accounts of the progress of the cattle plague in England and Europe. Amongst the most recent dispatches of this scourge are from our Consul at Liverpool up to the date of January 20 ult. Thus far he reports that 70,000 cattle have either died of this plague, or been killed after taking it. It is probable that additionally hundreds of cases are not reported at all, so that these figures by no means represent the full and actual loss. Vaccination is being tried as a preventive. Inasmuch as these scourges amongst the lower animals have come in some way to be regarded as forerunners of Asiatic Cholera, an item of plague amongst animals in this country will be read with interest. We are informed by a very intelligent physician from Grant County, Kentucky, that in his county large numbers of hogs are dying off from an epidemic, materially different from the disease familiary known in this country as "Hog Cholera." The animals sicken, become languid, helpless, refuse food, and linger a number of days before death. There is no diarrhœa, but frequently toward the termination of the case there is set up a dysentric discharge from the bowels. The general progress of the case resembling the typhoid fever of the human animal, and a *post mortem* examination revealing similar intestinal lesions.—*Cincinnati Lancet & Observer.*

---

Mortality for the Month of March.—The mortality report for the month of March, as copied from the books of the health-officer, is given below. The report is very gratifying when compared with the reports of the same month for the years 1864 and 1865. Last year in the month of March 279 deaths were reported. In March, 1864, the number was greater by 90 than now. Not one case of small-pox is reported, and the city is evidently entirely free from all kinds of epidemic disorders:—

CAUSES OF DEATH.

| | | | |
|---|---|---|---|
| Accidents, | 3 | Intermittent Fever, | 1 |
| Apoplexy, | 2 | Inflammation of Brain, | 3 |
| Asthma, | 1 | Inflammation of Bowels, | 5 |
| Bronchitis, | 1 | Inflammation of Lungs, | 9 |
| Brain Fever, | 5 | Inflammation of Liver, | 1 |
| Congestion of Brain, | 5 | Jaundice, | 3 |
| Congestion of Bowels, | 1 | Killed, | 1 |
| Congestion of Lungs, | 5 | Lung Fever, | 7 |
| Colds, | 2 | Liver Complaint, | 1 |
| Cancer, | 2 | Marasmus, | 1 |
| Consumption, | 31 | Measles, | 2 |
| Cramps, | 14 | Old Age, | 5 |
| Croup, | 12 | Pneumonia, | 4 |
| Convulsions, | 3 | Paralysis, | 4 |
| Childbirth, | 9 | Puerperal Fever, | 2 |
| Cerebro-Spinal Meningitis, | 4 | Suicide, | 3 |
| Cholera-Morbus, | 1 | Stillborn, | 9 |
| Cholera Infantum, | 2 | Scarlet Fever, | 12 |
| Decline, | 2 | Ship Fever, | 1 |
| Diphtheria, | 14 | Typhoid Fever, | 9 |
| Dropsy, | 6 | Typhus Fever, | 2 |
| Diarrhœa, | 3 | Teething, | 3 |
| Erysipelas, | 1 | Water on Brain, | 5 |
| Fever, | 2 | Worms, | 1 |
| Heart Disease, | 2 | Unknown, | 24 |
| Hemorrhage, | 1 | | |
| Hydrocephelas, | 1 | Total, | 254 |
| Intemperance, | 1 | | |

Ages of the Deceased.—Under 5 years, 122; over 5 and under 10 years, 14; over 10 and under 20, 11; over 20 and under 30, 34; over 30 and under

40, 28; over 40 and under 50, 12; over 50 and under 60, 7; over 60 and under 70, 9; over 70 and under 80, 8; unknown, 9. Total, 254.

NATIVITIES,

| | | | | | |
|---|---|---|---|---|---|
| Chicago, | 120 | Canada, | 3 | Prussia, | 1 |
| Other States, | 42 | Ireland, | 29 | Switzerland, | 3 |
| England, | 6 | Scotland, | 1 | Unknown, | 10 |
| Germany | 32 | Sweden, | 3 | | |
| Bohemia, | 1 | Norway, | 3 | Total | 254 |

DIVISIONS.

| | | | | | | | |
|---|---|---|---|---|---|---|---|
| North, | 62 | South, | 94 | West, | 98 | Total, | 254 |

